# Peroneus longus acute calcific tendinitis: a case report

**DOI:** 10.1186/1757-1626-2-7453

**Published:** 2009-06-01

**Authors:** George Mouzopoulos, Nikolaos Lasanianos, George Nikolaras, Mathaios Tzurbakis

**Affiliations:** 1st Orthopaedic Department of Evangelismos HospitalAthensGreece

## Abstract

Calcific tendinitis of the peroneus longus tendon is extremely rare, with only two cases described previously in the literature. Herein we discuss the diagnosis and management of a case with an acute calcific tendonitis of peroneus longus tendon.

## Introduction

Periarticular painful calcification is most commonly seen within the rotator cuff of the shoulder, although it may develop around the hip, wrist, elbow, knee, forefoot and neck [1]. The calcific deposits may be located within the tendon or in the soft tissues adjacent to the tendon or ligament near its attachment to the bone [2]. Calcific tendinitis is presented as an acute inflammatory reaction, associated with pain, local tenderness, swelling and redness. Misdiagnosis is common and leads to delay in treatment and recovery [2]. Herein we discuss the diagnosis and management of a case with an acute calcific tendonitis of peroneus longus tendon.

## Case presentation

A 32-year-old man (white, 75 kg, 182 cm and non-smoker) presented to our emergency department with a 24 hours history of increasing pain on the lateral aspect of his left foot. The patient was unable to bear weight on the affected side, but there was no history of trauma to the ankle. Also no medical history was mentioned.

Clinical examination revealed localised tenderness proximal to the base of the fifth metatarsal, under the surface of the cuboid, in the line of the peroneus longus tendon, associated with warmth and redness. Although there was a full range of motion at the ankle, sub-talar and midfoot joint, but foot passive forced supination and active pronation under resistance produced pain exacerbation.

**Figure 1.**
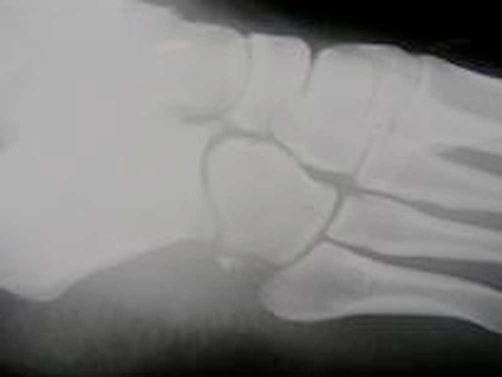
Calcific deposit at the lateral aspect of the foot.

A plain lateral radiograph showed homogeneous calcification at the lateral aspect of foot, under the surface of the cuboid in the line of the peroneus longus tendon (Figure 1). Laboratory examination showed white blood cells (WBC) = 12100/μl, C-reactive protein (CRP) = 3.1 mg/dl and erythrocytes segmentation rate (ESR) = 10 mm/h.

The patient was treated with a single dose of steroid injection (3 mg Betamethasone Sodium Phosphate + 3 mg Betamethasone Dipropionate mixed with 3 ml xylocaine) delivered at the area of maximum tenderness, following by ankle rest. After injection, the patient obtained dramatic relief from pain and was discharged the next day.

At review two weeks, 6 months and two years later the patient was remained without symptoms. A radiograph obtained after 6 months showed that the area of calcification had disappeared.

## Discussion

Calcific tendinitis of the peroneus longus tendon is extremely rare, with only two cases described previously in the literature [1,3].

The differential diagnosis of calcification in the region of peroneus longus includes avulsion fractures, soft tissue infection, myositis ossificans and sesamoid bones in the tendon. The diagnosis of calcific tendonitis is strongly suggested in the absence of foot trauma, systemic septic symptoms, cerebrovascular injury and normal radiographic view of contralateral foot. Also a comet-tail appearance of the calcific deposit on plain x-rays suggests that it lies within a tendon [4].

The exact mechanisms of the origin of the calcium deposits are not clearly understood. Healing of tendon injury by calcification, local stress necrosis directly or through fatty-acid and soap intermediaries and local hypoxia secondary to either mechanical or vascular factors have been proposed as causative factors [4].

Brinsden and Wilson suggested that non-operative management, consisted of NSAID treatment and rest could lead to early clinical improvement [3]. Cox and Paterson mentioned that steroid injection around calcific deposit is associated with immediate relief [1]. However, surgical debridement of intratendinous deposits could be helpful in difficult cases especially when conservative treatment is without effect [3].

## List of abbreviations

CRP: C-reactive protein
NSAID: Non-steroidal anti-inflammatory drugs
SER: Erythrocytes segmentation rate
WBC: White blood cells
